# Effect of SGLT2 Inhibitors on Type 2 Diabetes Mellitus With Non-Alcoholic Fatty Liver Disease: A Meta-Analysis of Randomized Controlled Trials

**DOI:** 10.3389/fendo.2021.635556

**Published:** 2021-06-17

**Authors:** Qiong Wei, Xinyue Xu, Li Guo, Jia Li, Ling Li

**Affiliations:** ^1^ Department of Endocrinology, Zhongda Hospital, School of Medicine, Southeast University, Nanjing, China; ^2^ Institute of Pancreas, Southeast University, Nanjing, China; ^3^ Department of Ultrasonography, Zhongda Hospital, Medical School, Southeast University, Nanjing, China

**Keywords:** sodium-glucose cotransporter 2 inhibitors, type 2 diabetes mellitus, liver proton density fat fraction, visceral fat mass area, non-alcoholic fatty liver disease

## Abstract

**Objective:**

Clinical trials showed that sodium-glucose cotransporter 2 (SGLT2) inhibitors can improve non-alcoholic fatty liver disease (NAFLD). In this work, a meta-analysis of randomized controlled trials was conducted to evaluate the effect of SGLT2 inhibitors on type 2 diabetes mellitus (T2DM) with NAFLD.

**Methods:**

PubMed, Embase, Web of Science, and Cochrane Libraries were used for the systematic literature review to determine eligible studies. A randomized effect model was adapted to perform a meta-analysis on these eligible studies to estimate the combined effect sizes. Differences were expressed as the weighted average difference (WMD) of the continuous results and the 95% confidence interval (CI).

**Results:**

Ten randomized controlled trials with 573 participants were included. SGLT2 inhibitors significantly reduced the levels of alanine transaminase (WMD -5.36 [95% CI: -8.86, -1.85], p = 0.003) and Aspartate Transaminase (WMD -2.56 [95% CI: -3.83, -1.29], p <0.0001). In terms of body composition, liver proton density fat fraction (WMD -2.20 [95% CI: -3.67, -0.74], p = 0.003), visceral fat mass area (WMD -20.71 [95% CI: -28.19, -13.23], p <0.00001), subcutaneous fat areas (WMD -14.68 [95% CI: -26.96, -2.40], p = 0.02) were also significantly reduced.

**Conclusion:**

SGLT2 inhibitors can remarkably reduce hepatic enzymes, hepatic fat and improve body composition. Thus, they may become a new treatment option for NAFLD.

**Systematic Review Registration:**

PROSPERO, identifier CRD42020215570.

## Introduction

Non-alcoholic fatty liver disease (NAFLD) is the most common liver disease, and its prevalence continues to increase worldwide. T2DM and NAFLD have a close relationship, and approximately 80% of patients with type 2 diabetes mellitus (T2DM) have NAFLD (1). NAFLD is a complication and a risk factor of T2DM. Its pathogenesis is complex, including insulin resistance (a common key factor in T2DM and NAFLD), oxidative stress, and mitochondrial dysfunction (2), which causes non-alcoholic steatohepatitis (NASH) to aggravate insulin resistance. Insulin resistance is a common key factor in the occurrence of T2DM and NAFLD (3, 4). NAFLD is usually accompanied by various complications, such as cardiovascular disease and chronic kidney diseases (5, 6), thereby seriously affecting the life expectancy of patients with T2DM. Therefore, early intervention and treatment of T2DM with NAFLD are required. Given the lack of approved drug therapies for NAFLD, medicines for related complications such as diabetes are being investigated for possible liver-related benefits. Although the insulin sensitizer pioglitazone can play a antihyperglycemic role and improve liver function, it is used cautiously in clinical practice because of its problematic safety in long-term use and side effects such as weight gain and edema (7).

Sodium-glucose cotransporter 2 (SGLT2) inhibitors are novel oral antihyperglycemic drugs that have received attention due to their unique mechanism of inhibiting glucose reabsorption in the proximal renal tubules and increasing urinary glucose excretion (8). This type of antihyperglycemic method does not depend on insulin and reduces body weight (9). Clinical trials reported that SGLT2 inhibitors can improve NAFLD and reduce Aspartate Transaminase (AST) and liver fat in patients with T2DM and NAFLD (10). However, contrary opinions have been held in some studies (11). The previous studies summarized the effects of SGLT2 inhibitors on hepatic fat and hepatic enzymes, but failed to assess the effects on liver fibrosis. Therefore, whether SGLT2 inhibitors affect liver fibrosis requires further discussion. Newer, larger sample and longer term studies with relatively complete data need to be included, and the preliminary conclusion needs to be updated. For this purpose, we once again reviewed trials on SGLT2 inhibitors to reach a more comprehensive and reliable conclusion.

## Method

### Search Strategy

Related articles as of July 2020 were searched from PubMed, Embase, Web of Science, and Cochrane libraries by using the following subject terms: (Sodium-Glucose Transporter 2 Inhibitors OR SGLT2 Inhibitors OR SGLT 2 Inhibitors OR SGLT2Inhibitors OR Sotagliflozin OR Dapagliflozin OR Empagliflozin OR Canagliflozin OR Luseogliflozin OR Ipragliflozin OR Canagliflozin OR Ertugliflozin OR Gliflozins) AND (Non-alcoholic Fatty Liver Disease OR NAFLD OR Nonalcoholic Fatty Liver Disease OR Fatty Liver, Nonalcoholic OR Fatty Livers, Nonalcoholic OR Liver, Nonalcoholic Fatty OR Livers, Nonalcoholic Fatty OR Nonalcoholic Fatty Liver OR Nonalcoholic Steatohepatitis OR Nonalcoholic Steatohepatitides OR Steatohepatitides, Nonalcoholic OR Steatohepatitis, Nonalcoholic), no language restrictions. Two reviewers (Xinyue Xu and Li Guo) independently selected the relevant articles according to the title and abstract and then screened the full text to determine whether it meets the inclusion or exclusion criteria. References to all eligible trials, meta-analyses, and related reviews were also reviewed to avoid missing any articles. All search results were downloaded into EndNote (version X9, Thomson Reuters, Philadelphia, PA, USA) to eliminate duplication, and the searched citations were merged to simplify the screening process.

### Eligibility Criteria

All randomized controlled clinical trials (RCTs) evaluating the effects of SGLT2 inhibitors on patients with T2DM and NAFLD were included in the meta-analysis. Inclusion criteria were as follows: (a) the population was patients with T2DM and NAFLD aged 18–75 years; HbA1c: 6%–12%, fatty liver confirmed by imaging examination (ultrasound or computed tomography); and alcohol intake should not exceed 140 g/week for women and 210 g/week for men. Other causes of liver disease (such as viral hepatitis, autoimmune hepatitis, and primary biliary cirrhosis) were excluded. (b) Therapeutic intervention includes various types of SGLT2 inhibitors, and the control group has sufficient information about baseline and post-treatment in the study report, such as glycosylated hemoglobin A1c (HbA1c), fasting plasma glucose (FPG), homoeostasis model assessment of insulin resistance (HOMA-IR), alanine transaminase (ALT), Aspartate Transaminase (AST), fibrosis 4 (FIB-4) index, liver proton density fat fraction (PDFF), visceral fat area (VFA), subcutaneous fat areas (SFA), and body weight. (c) The full text was provided, and the study was designed as a randomized controlled trial. Exclusion criteria includes the following: (a) animal studies; (b) pregnant or breastfeeding women; (c) non-randomized controlled trials; and (d) reviews, conference abstracts, reviews, meta-analysis, incomplete article results, and case reports.

### Data Extraction and Assessment for Study Quality

The following relevant data were extracted from each study: first author, year of publication, sample size, intervention (type and dose of SGLT2 inhibitors), drug in the comparison group, follow-up time, and patient baseline information.

Two reviewers (Xinyue Xu and Li Guo) independently assessed the quality of RCTs using the Cochrane Risk of Bias Tool, which included the following seven criteria: random sequence generation (selection bias), allocation concealment (selection bias), blinding of participants and personnel (performance bias), blinding of outcome assessment (detection bias), incomplete outcome data (attrition bias), reporting bias selective reporting (reporting bias), and other biases (certain biases not indicated above but influence the results). As per the recommendations of the Cochrane Handbook, each project was evaluated as a “low risk,” “high risk,” or “unclear risk” risk of bias.

### Statistical Analysis

RevMan 5.4 software provided by Cochrane was used for analysis, and the measurement data were all presented as mean and standard deviation (SD). Outcomes were compared using weighted mean difference (WMD), and 95% confidence interval (CI) was used for each effect. I^2^ statistics was used to assess the heterogeneity of the studies. Studies with an I^2^ statistic of 25%–50% were considered as low heterogeneity, those with an I^2^ of 50%–75% as moderate heterogeneity, and those with an I^2^ of > 75% as high heterogeneity. Random effects models were used in all studies. According to the characteristics of the study, subsequent subgroup analysis or sensitivity analysis was conducted to explain the reasons for heterogeneity. Forest plot was drawn by RevMan defaults to adverse events. When the 95% CI horizontal line of a study does not intersect the forest plot invalid line and falls to the left of the invalid line, it can be considered that the mean of the indicator in the SGLT2 inhibitor group was less than that in the control group, that is, the experimental factors in the SGLT2 inhibitors group reduced the occurrence of adverse events. Hence, the experimental factors were beneficial and effective. P values of less than 0.05 were considered statistically significant.

## Results

### Literature Search

**Figure 1:**
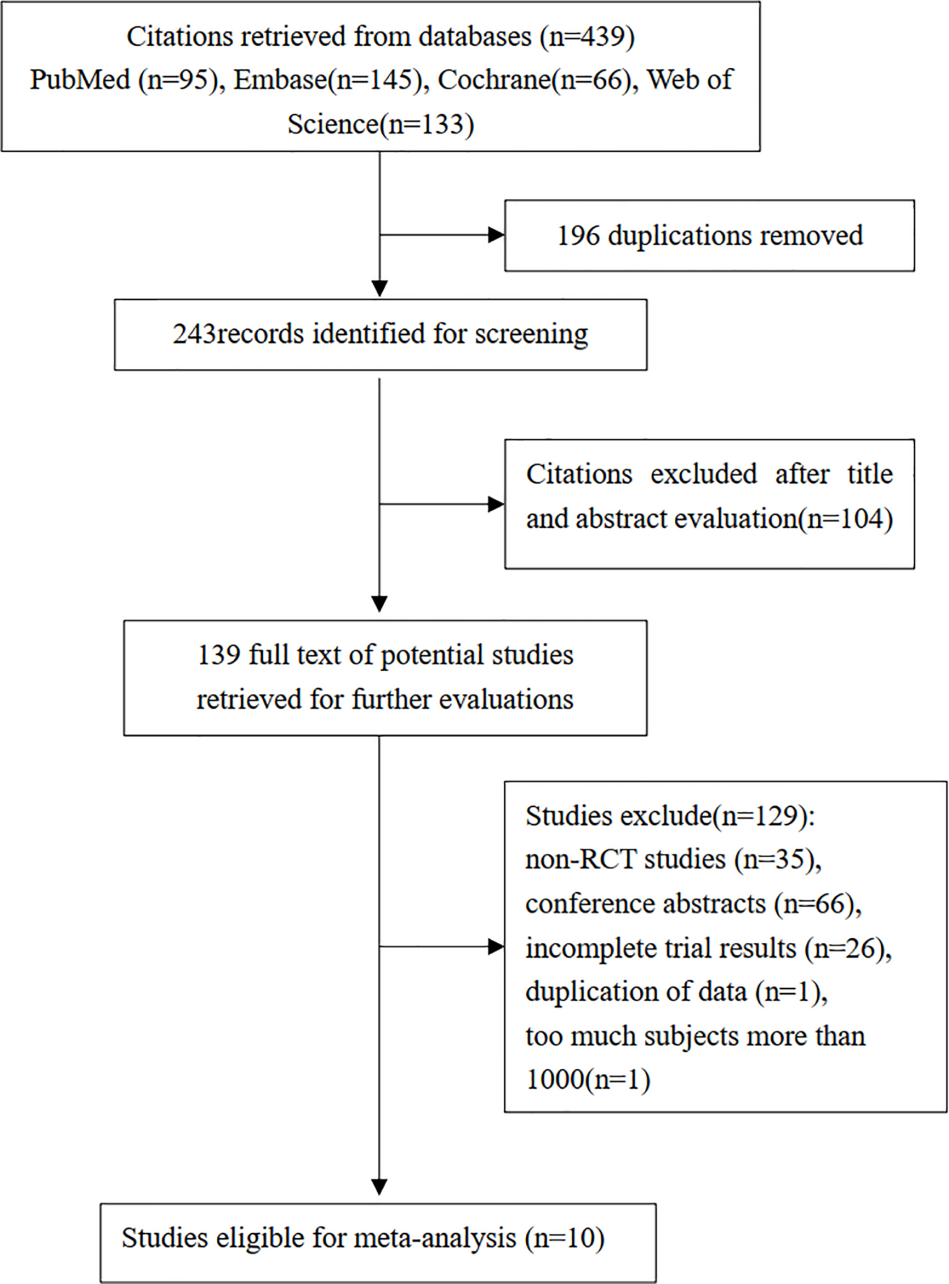
Summary of study identification, inclusion, and exclusion.

The literature search identified 439 studies, 95 of which were from PubMed, 145 from Embase, 66 from the Cochrane Library, and 133 from the Web of Science. A total of 196 duplicate papers were excluded, 104 papers were removed based on title and abstract, and from 139 studies were evaluated further, 129 studies were excluded for the following reasons: (a) non-RCT studies (n=35), (b) conference abstracts (n=66), (c) incomplete trial results (n=26), (d) exclusion due to more than 1000 subjects to avoid overweighting (n=1), and (e) duplication of data (n=1). The final 10 eligible studies were included in the final meta-analysis (10–19). **Figure 1** describes the study selection flowchart in detail.

### Study Characteristics and Quality Assessment

**Figure 2:**
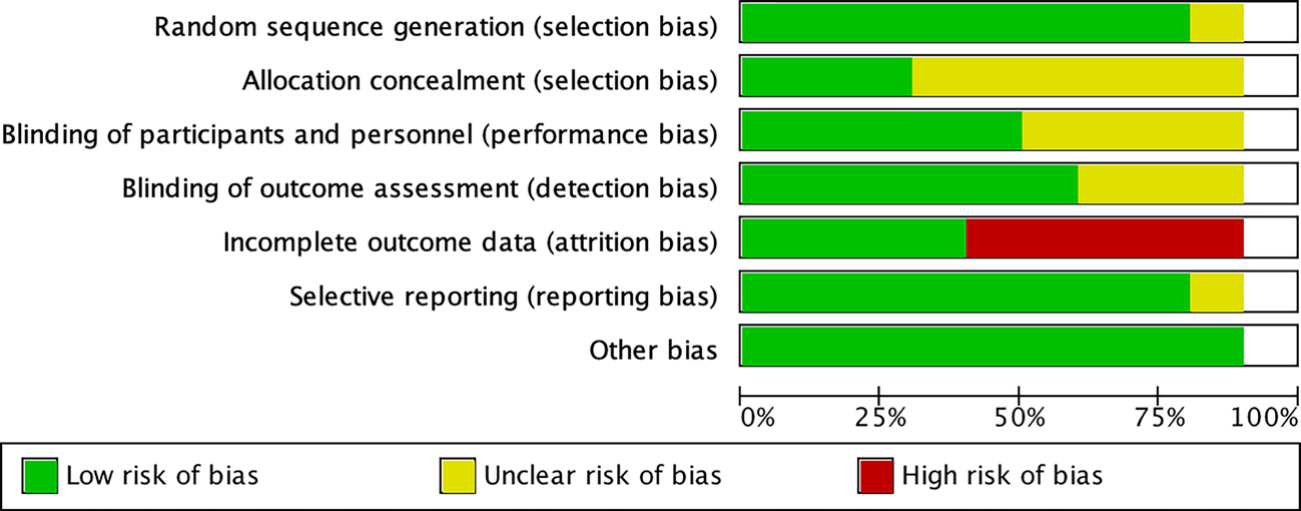
Quality evaluation chart of included studies.

The included research characteristics are shown in **Table 1**. The articles presented 10 trials involving 573 subjects (295 SGLT2 inhibitors subjects and 278 control subjects) published between 2017 and 2020. Four multicenter studies were included, and five were from Japan, four studies were from Germany, South Korea, Sweden, India, the remaining one study was conducted at 87 centers in Germany, the Czech Republic, Hungary, Mexico, Poland, Romania, Russia, Sweden, the UK and the United States. Many types of SGLT2 inhibitors were included in the intervention group, including empagliflozin (two studies), dapagliflozin (five studies), Ipragliflozin (three studies), and Luseogliflozin (one study). One study used empagliflozin or dapagliflozin. For the control group, the intervention drugs were as follows: metformin, pioglitazone, sitagliptin, and Glucagon-like peptide-1 receptor agonist, glimepiride. The duration of follow-up ranged from 12 weeks to 52 weeks. Urinary tract infections were reported in three studies, hypoglycemia was reported in one study, and arthralgia in large joints was reported in one study. Four studies reported no serious adverse effects, and three studies reported no adverse events. In summary, the SGLT2 inhibitors were safe. According to the Cochrane Risk Bias Tool, all studies are parallel grouping studies with high quality. Quality assessment results for the included studies are summarized in **Figure 2**.

**Table 1:** Demographic and clinical characteristics of included studies.

Source	Sample(F)	Age(years)	Duration(years)	Trial Duration	Agent (Daily Dosage)	Comparator (Dosage)
Aso Y (19)	57 (23)	55.0 ± 8.6	NR	24 weeks	Standard-hypoglycemic treatment	Standard-hypoglycemic treatment
					+ dapagliflozin 5 mg	
Bando Y (18)	62 (22)	55.1 ± 8.6	9.6 ± 4.5	12 weeks	Continued-hypoglycemic treatment	Continued-hypoglycemic treatment
					+ ipragliflozin 50 mg	
Eriksson JW (17)	42 (9)	65 ± 36	6.6 ± 5.1	12 weeks	Dapagliflozin 10 mg	Placebo
Han E (11)	45 (17)	53.9 ± 10.9	9.4 ± 5.8	24 weeks	Metformin + pioglitazone	Metformin + pioglitazone
					+ ipragliflozin 50 mg	
Ito D (16)	66 (34)	58.2 ± 10.9	9.1 ± 5.8	24 weeks	Ipragliflozin 50 mg	Pioglitazone 15-30 mg/day
Johansson L (15)	82 (41)	58.0 ± 9.0	6.4 ± 6.0	52 weeks	metformin + saxagliptin 5 mg + placebo + dapagliflozin 10mg	metformin + glimepiride 1-6 mg + placebo
Kinoshita T (10)	98 (45)	59 ± 1	7.2 ± 0.5	28 weeks	Dapagliflozin 5 mg	Pioglitazone 7.5–15 mg/day
Kuchay MS (14)	50 (20)	65.3 ± 6.23	NR	20 weeks	Standard-hypoglycemic treatment	Standard-hypoglycemic treatment
					+ empagliflozin 10 mg	
Mittag-Roussou V (13)	39 (19)	57.7 ± 10.9	NR	6 months	Dapagliflozin 5 – 10 mg empagliflozin 10 – 25 mg	Exenatide 10 – 20 μg or liraglutide 0.6 - 1.8 mg or dulaglutide 0.75 - 1.5 mg/week.
Shibuya T (12)	32 (14)	56.5 ± 11.68	9.6 ± 1.0	6 months	Luseogliflozin 5 mg	Metformin 1500 mg/day

F, Female; NR, No report.

### Effect of SGLT2 Inhibitors on Glycemic Indices

#### Glycosylated Hemoglobin A1c

**Figure 3:**
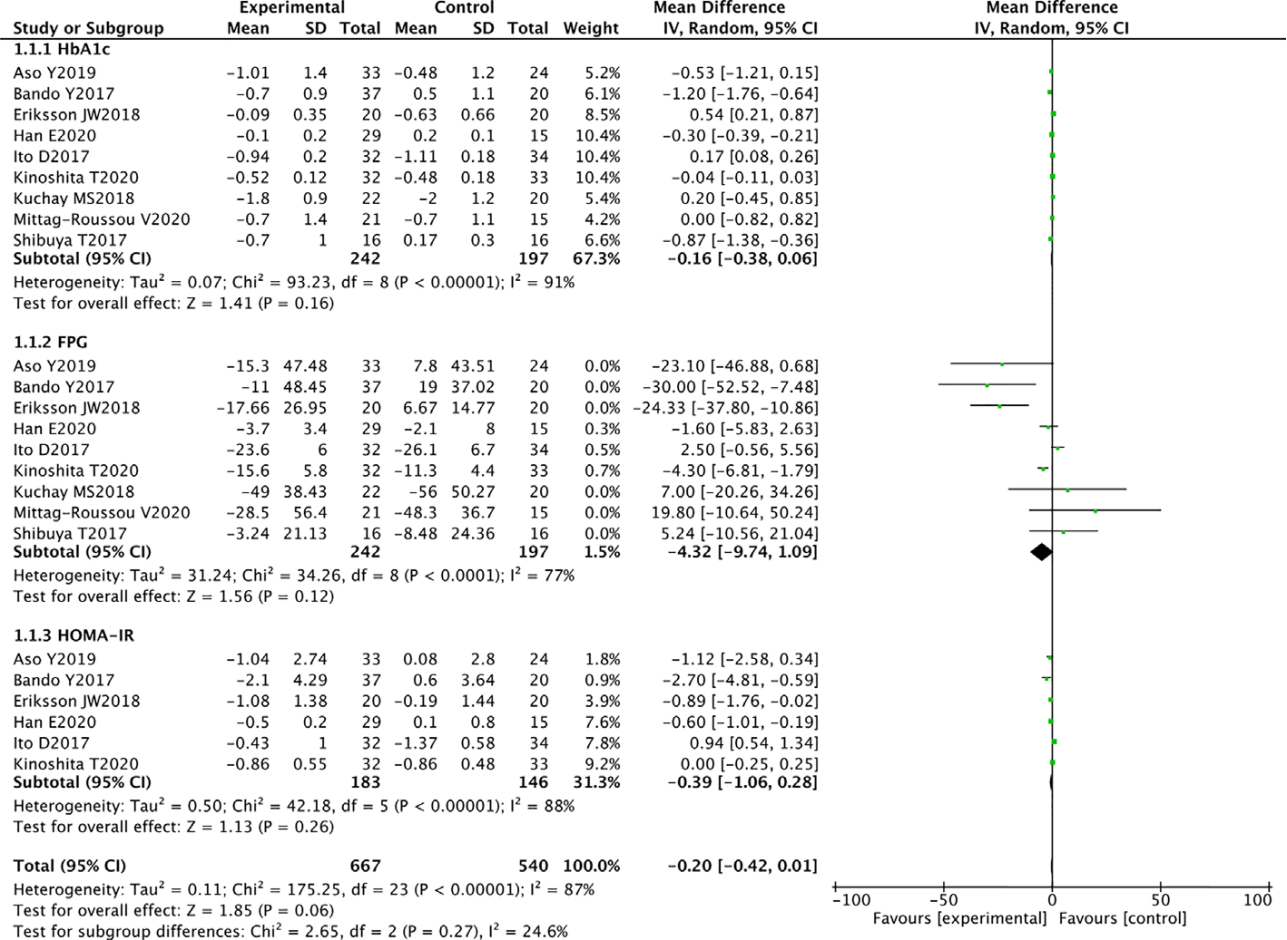
Meta-analysis of the effect of SGLT2 inhibitors on glycemic indices compared with the control group.

Nine RCTs reported HbA1c levels in 242 SGLT2 inhibitors users and 197 non-users. Compared with other antihyperglycemic drugs, SGLT2 inhibitors were more likely to reduce HbA1c, but the difference was not statistically significant (WMD-0.16 [95%CI: -0.38, 0.06], P = 0.16). I^2^ = 91%, heterogeneity was greater, and no heterogeneity analysis was conducted because the two groups were not statistically different (**Figure 3**).

#### Fasting Plasma Glucose

In 9 RCTs, a pooled study of 439 participants showed that SGLT2 inhibitors were likely to reduce FPG, but no statistical difference was observed between the two groups (WMD -4.32 [95% CI: -9.74, 1.09], p = 0.12). The results are highly heterogeneous (P <0.00001, I^2^ = 77%) (**Figure 3**).

#### Homeostatic Model Assessment of Insulin Resistance

Six studies evaluated the effects of SGLT2 inhibitors on HOMA-IR. Overall analysis showed that SGLT2 inhibitors did not significantly reduce HOMA-IR compared with other antihyperglycemic agents (WMD -0.39 [95%CI: -1.06, 0.28], P = 0.26). These findings suggest that SGLT2 inhibitors are not superior over other drugs in improving insulin resistance. Significant heterogeneity was observed between studies (P < 0.00001, I^2^ = 88%) (**Figure 3**).

High evidence RCTs in this meta-analysis revealed that SGLT2 inhibitors reduced the HBA1c, FPG, and HOMA-IR levels. However, some studies included other effective ﻿antidiabetic drugs, thus influencing the final data analysis and resulting in high heterogeneity in the meta-analysis.

### Effect of SGLT2 Inhibitors on Liver Functions

#### Alanine Transaminase

**Figure 4:**
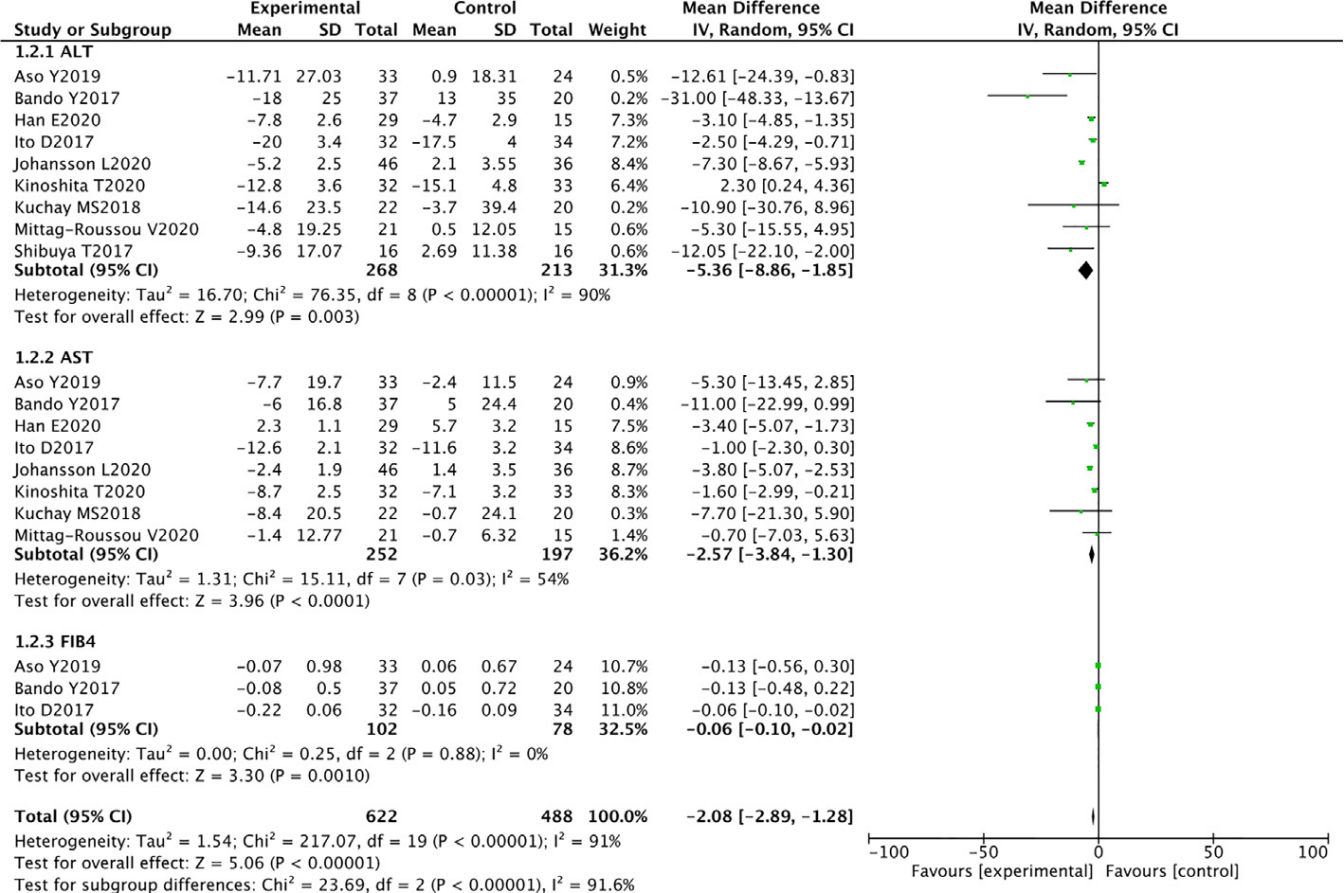
Meta-analysis of the effect of SGLT2 inhibitors on liver function compared with the control group.

Nine RCTs reported ALT levels in 268 SGLT2 inhibitors users and 213 non-users. The meta-analysis showed that SGLT2 inhibitors significantly reduced the ALT levels in patients with NAFLD compared with other drugs (WMD -5.36 [95% CI: -8.86, -1.85], p = 0.003). Significant heterogeneity was found between studies (p < 0.00001, I^2^ = 90%) (**Figure 4**).

#### Aspartate Transaminase

Eight RCTs reported AST levels in 252 SGLT2 inhibitors users and 197 non-users. The meta-analysis showed that SGLT2 inhibitors significantly reduced the AST levels in patients with NAFLD (WMD -2.57 [95% CI: -3.84, -1.30], p < 0.0001). Moderate heterogeneity was observed between studies (p = 0.03, I^2^ = 54%) (**Figure 4**).

#### Fibrosis-4 Index for Liver Fibrosis

Three RCTs evaluated the effect of SGLT2 inhibitors on FIB-4. Compared with other antihyperglycemic drugs, SGLT2 inhibitors can significantly reduce FIB-4 (WMD -0.06 [95% CI: -0.10, -0.02], p = 0.0010). No heterogeneity was observed between studies (p = 0.88, I^2^ = 0%) (**Figure 4**).

These randomized controlled trials strongly support the use of SGLT2 inhibitors to reduce ALT and AST levels and improve liver fibrosis in patients with T2DM and NAFLD.

### Effect of SGLT2 Inhibitors on Body Composition

#### Body Weight

**Figure 5:**
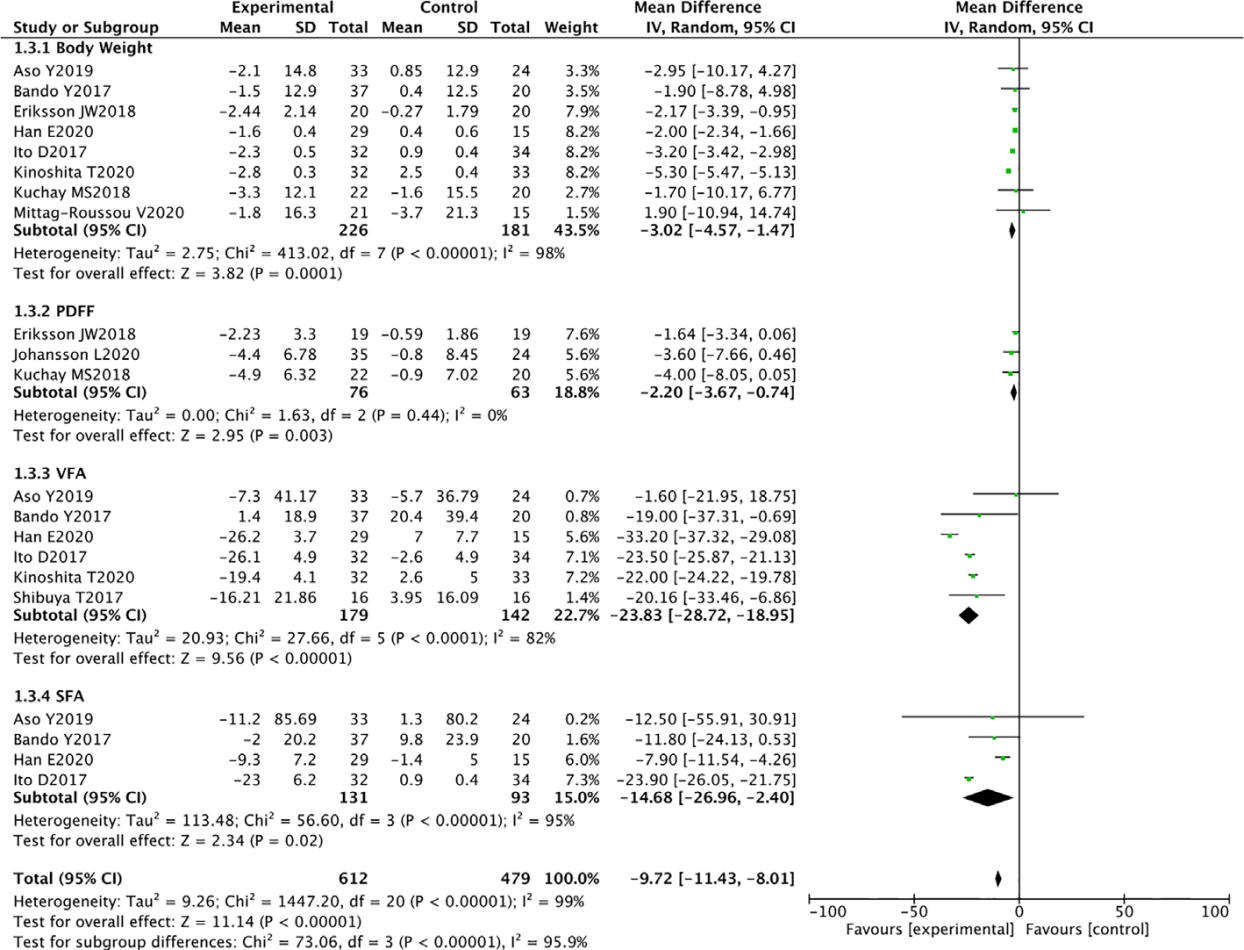
Meta-analysis of the effect of SGLT2 inhibitors on body composition compared with the control group.

Eight RCTs reported the weight of 226 SGLT2 inhibitors users and 181 non-users. Compared with other antihyperglycemic drugs, SGLT2 inhibitors can significantly reduce body weight (WMD -3.02 [95% CI: -4.57, -1.47], p = 0.0001). Significant heterogeneity was found between studies (P < 0.00001, I^2^ = 98%) (**Figure 5**).

#### Proton Density Fat Fraction

PDFF is a quantitative biomarker that is based on MRI and can accurately estimate liver fat content. Three RCTs reported PDFF in 76 SGLT2 inhibitors users and 63 non-users. SGLT2 inhibitors significantly reduced PDFF compared with other ﻿antidiabetic drugs (WMD -2.20 [95% CI: -3.67, -0.74], p = 0.003). No heterogeneity was observed between studies (p = 0.44, I^2^ = 0%) (**Figure 5**).

#### Visceral Fat Area

Six RCTs reported the measurement of VFA using DXA in 179 SGLT2 inhibitors users and 142 non-users. The results showed that SGLT2 inhibitors significantly reduced VFA compared with other ﻿antidiabetic drugs (WMD -23.83 [95% CI: -28.72, -18.95], p < 0.00001). Significant heterogeneity was found between studies (p < 0.0001, I^2^ = 82%) (**Figure 5**).

#### Subcutaneous Fat Areas

Four RCTs reported the measurement of SFA in 131 SGLT2 inhibitors users and 93 non-users using DXA. The results showed that SGLT2 inhibitors significantly reduced SFA compared with other ﻿antidiabetic drugs (WMD -14.68 [95% CI: -26.96, -2.40], p = 0.02). Significant heterogeneity was noted between studies (p < 0.00001, I^2^ = 95%) (**Figure 5**).

These randomized controlled trials provide a strong support for the use of SGLT2 inhibitors to reduce weight, liver fat, VFA, and SFA in patients with T2DM and NAFLD.

### Sensitivity Analyses and Subgroup Analyses

**Figure 6:**
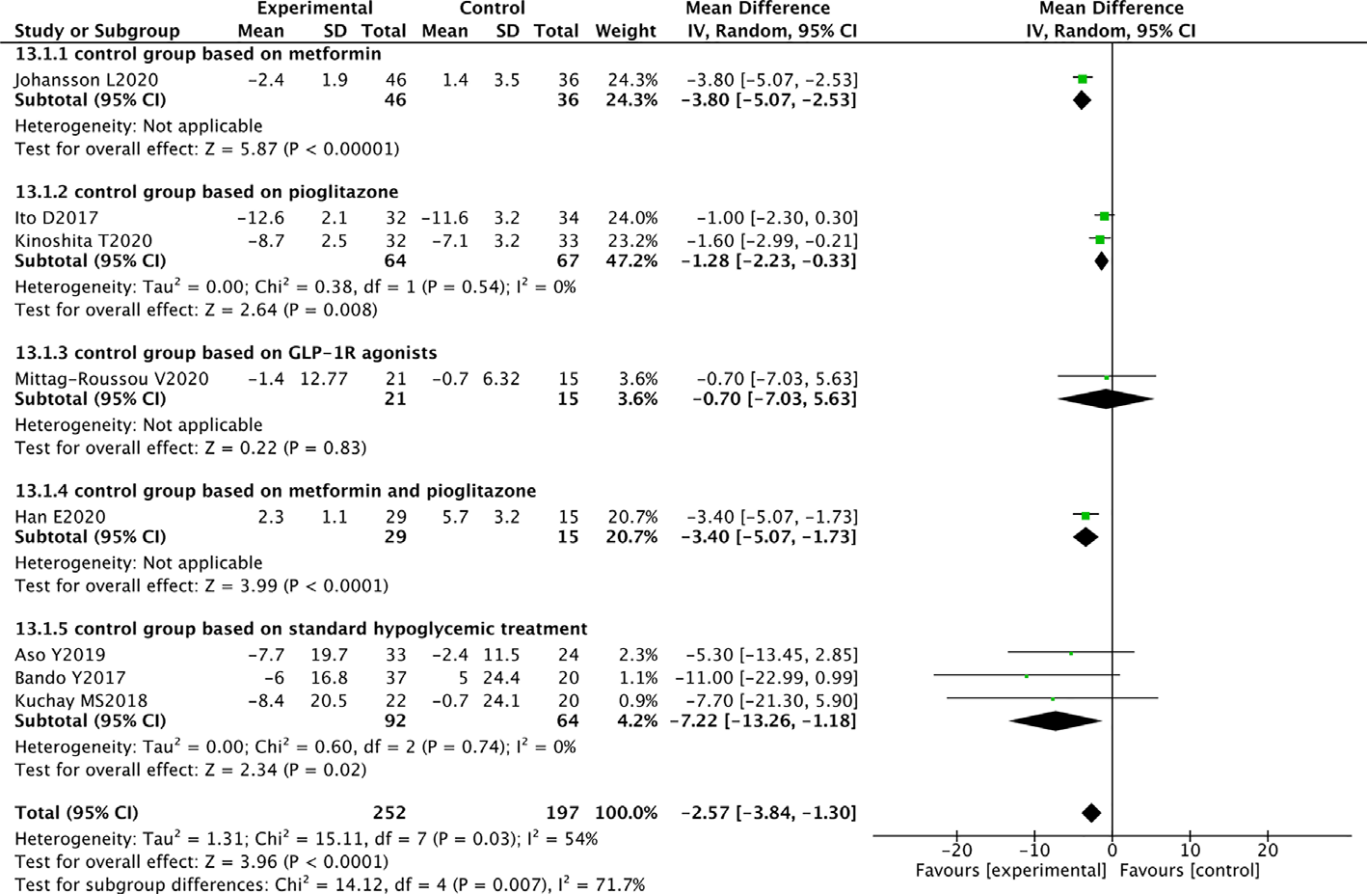
Meta-analysis of AST level comparisons between SGLT2 inhibitors and the control group based on the control.

**Figure 7:**
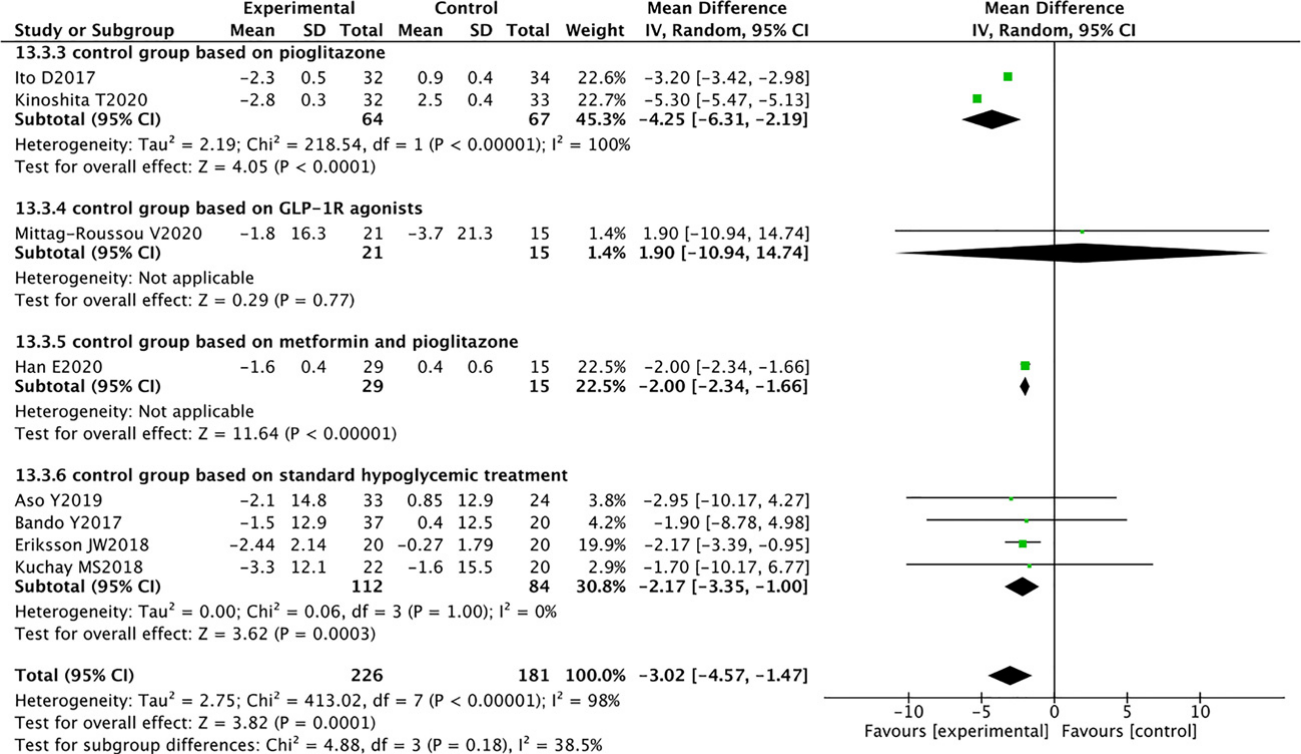
Meta-analysis of body weight level comparisons between SGLT2 inhibitors and the control group based on the control.

Sensitivity analyses were performed to identify the sources of heterogeneity. When pioglitazone was excluded from the control group, the heterogeneity was significantly reduced. For example, in AST analysis, when the three RCTs of Han, Ito, and Kinoshita were excluded, the heterogeneity changed from 54% to 0%. In the weight analysis, when these three RCTs were excluded, the heterogeneity changed from 89% to 0%. Therefore, further subgroup analysis was performed based on the differences in the control group. The results suggested that SGLT2 inhibitors reduced the AST levels in patients with NAFLD and had superior effect over pioglitazone (WMD -1.28 [95% CI: -2.23, -0.33], p = 0.008) and metformin (WMD -3.80[95% CI: -5.07, -2.53], p < 0.00001) (**Figure 6**). In terms of weight reduction, SGLT2 inhibitors were superior over pioglitazone (WMD -4.25 [95% CI: -6.31, -2.19], p < 0.0001) (**Figure 7**).

### Publication Bias

Given that only 10 RCTs were included in this meta-analysis, the number of articles was small, and publication bias tests were not performed.

## Discussion

This meta-analysis of 10 RCTs aimed to evaluate the effect of SGLT-2 inhibitors in patients with T2DM and NAFLD. The ability of SGLT-2 inhibitors to control glycemia and improve fatty liver was investigated. Results showed that SGLT-2 inhibitors were superior over other antihyperglycemic drugs used in these RCTs in improving liver enzymes, reducing liver fat, reducing body weight, and improving liver fibrosis. No statistically significant differences were found in their activities of lowering blood glucose or improving insulin resistance.

As a chronic disease, NAFLD has become an important health problem. NAFLD, coronary heart disease, hypertension, and atherosclerosis are metabolic syndromes with insulin resistance as their common mechanism. T2DM combined with NAFLD further disrupts glucose metabolism and increases the incidence of NASH. The coexistence of high glucotoxicity and lipotoxicity substantially increases the incidence of diabetic macrovascular events and accelerates the transformation of fatty liver into cirrhosis and even liver cancer. NAFLD is often accompanied by oxidative stress, dyslipidemia, inflammation, and insulin resistance. Therefore, its treatment should focus on correcting these disorders.

SGLT2 inhibitors positively affect chronic diseases, including diabetes, obesity, cardiovascular disease (20), and kidney disease (21). This meta-analysis confirmed that SGLT2 inhibitors can reduce liver fat, lower ALT, AST, and FIB-4 levels and alleviate NAFLD. Animal experiments revealed that SGLT2 inhibitors can improve fatty liver by reducing lipid production, enhancing insulin resistance, and alleviating endoplasmic reticulum stress (22). However, the pathophysiological mechanisms of these effects on NAFLD are controversial. The tissue characteristics of NAFLD are predominantly hepatic lipid accumulation, which is caused by an imbalance between hepatic triglyceride synthesis and fatty acid oxidation (23). SGLT2 inhibitors induce a metabolic shift from carbohydrate oxidation to fatty acid oxidation, thus possibly prevent lipid accumulation by increasing fatty acid oxidation in adipose tissues and the liver. In addition, they can reduce energy by excreting glucose in the urine (24). This energy loss may promote β-oxidation in liver and visceral fat, induce liver fat metabolism, and reduce VFA. The latter decreases the transport of fatty acids from adipose tissues to the liver, corrects hyperinsulinemia, and increases adiponectin levels. The adenosine monophosphate-activated protein kinase pathway is activated by adiponectin, which inhibits fat formation and accelerates the oxidation of fatty acids in the liver (25). The main pathological status of patients with NAFLD is insulin resistance (3), Excessive insulin levels promote lipogenesis, leading to liver steatosis. SGLT2 inhibitors lower the blood glucose and gradually correct hyperinsulinemia (26) while improving insulin resistance and reducing hepatic lipogenesis. Additional pathogenesis of NAFLD includes oxidative stress, mitochondrial dysfunction, and endoplasmic reticulum homeostasis. SGLT2 inhibitors directly inhibited the enhanced expression of dipeptidyl peptidase‐4 in the liver (19), reduced the plasma FGF21 levels (27, 28), and improved the mitochondrial function or reduced endoplasmic reticulum stress in the liver.

Pioglitazone can reduce hepatic enzymes and hepatic fat. In this meta-analysis, SGLT2 inhibitors were superior in reducing AST compared with pioglitazone, and the difference was statistically significant. Pioglitazone may also increase VFA, SFA, and body weight, which is not conducive to the long-term prognosis of patients with NAFLD (10). This meta-analysis showed that compared with glucagon-like peptide-1 receptor (GLP-1R) agonists, SGLT-2 inhibitors can improve AST. However, the difference was not statistically significant. Only one study and the small number of subjects included in this study severely limited our ability to analyze the differences between SGLT-2 inhibitors and GLP-1R agonists in affecting the liver (13). Moreover, the use of GLP-1R agonists is limited due to their disadvantages such as severe gastrointestinal discomfort and the need for subcutaneous injections.

This meta-analysis showed that SGLT2 inhibitors were not superior over other antihyperglycemic drugs in lowering blood glucose but effectively improved NAFLD. Considering the mechanism of SGLT2 inhibitors on NAFLD, they may not be dependent on lowering blood glucose to improve fatty liver. This finding may provide a new idea for the treatment of NAFLD patients without T2DM.

The highlight of this meta-analysis was the comprehensive evaluation of the effects of SGLT2 inhibitors on patients with T2DM and NAFLD in terms of blood glucose control, improvement of liver enzymes, and influence on body composition. This work provided evidence for the use of SGLT2 inhibitors in these patients. However, the following are the limitations of this article: (a) only a few randomized controlled trials met the conditions, and most RCTs have small sample sizes and thus produce insignificant results. Additional RCTs are needed to further validate the current results. (b) The majority of the RCTs included in this study were from Asia. Ethnic differences and Asian-specific lifestyles may have influenced the results. (c) The included studies had short follow up periods with the longest at only 52 weeks. In addition, the long-term prognosis for SGLT2 inhibitors is unclear, thus requiring continued follow-up. (d) NAFLD diagnosis was based on imaging, and no histological examination was conducted. Therefore, the effect of SGLT2 inhibitors on T2DM with NAFLD was not histologically evaluated.

## Conclusion

For non-alcoholic fatty liver disease, SGLT2 inhibitors are more effective in reducing AST, ALT, FIB-4, PDFF, VFA, SFA, and weight levels than other antihyperglycemic drug; however, their decreasing effect on HbA1c, FPG, and HOMA-IR levels was not superior over other antihyperglycemic drugs. The improvement of NAFLD by SGLT2 inhibitors provides a new treatment option for patients with T2DM and NAFLD.

## Data Availability Statement

The original contributions presented in the study are included in the article/supplementary material. ﻿Further inquiries can be directed to the corresponding authors.

## Author Contributions

QW designed the study and reviewed the manuscript. XX collected the data, analyzed the data, and wrote the manuscript. LG collected the data and analyzed the data. JL contributed to the introduction and reviewed the manuscript. LL designed the study and contributed to the introduction and the discussion. ﻿All authors contributed to the article and approved the submitted version.

## Conflict of Interest

The authors declare that the research was conducted in the absence of any commercial or financial relationships that could be construed as a potential conflict of interest.
